# Quality of life and emotional distress in sarcoma patients diagnosed during COVID-19 pandemic: a supplementary analysis from the SarCorD study

**DOI:** 10.3389/fpsyg.2023.1078992

**Published:** 2023-06-02

**Authors:** Concetta Elisa Onesti, Sabrina Vari, Denise Minghelli, Francesca Nardozza, Barbara Rossi, Francesca Sperati, Elisa Checcucci, Wioletta Faltyn, Maria Cecilia Cercato, Antonella Cosimati, Francesca Salvatori, Roberto Biagini, Gennaro Ciliberto, Virginia Ferraresi, Gabriella Maggi

**Affiliations:** ^1^Sarcomas and Rare Tumors Unit, IRCCS Regina Elena National Cancer Institute, Rome, Italy; ^2^Psychology Unit, IRCCS Regina Elena National Cancer Institute, Rome, Italy; ^3^UOSD Clinical Trial Center, Biostatistics and Bioinformatics, IRCCS Regina Elena National Cancer Institute, Rome, Italy; ^4^Oncological Orthopaedics Unit, IRCCS Regina Elena National Cancer Institute, Rome, Italy; ^5^UOSD Clinical Trial Center, Biostatistics and Bioinformatics, San Gallicano Dermatological Institute IRCCS, Rome, Italy; ^6^Epidemiology and Tumor Registry Unit, IRCCS Regina Elena National Cancer Institute, Rome, Italy; ^7^Digital Library “R. Maceratini”, IRCCS Regina Elena National Cancer Institute, Rome, Italy; ^8^University of Rome “Sapienza”, Rome, Italy; ^9^Scientific Direction, IRCCS Regina Elena National Cancer Institute, Rome, Italy

**Keywords:** COVID-19, sarcoma, quality of life, bone sarcoma, soft tissue sarcoma, aggressive benign musculoskeletal disease

## Abstract

**Background:**

The COVID-19 outbreak had a negative psychological impact on cancer patients. In this study, we analyzed emotional distress and quality of life in patients diagnosed with sarcoma during the first year of the pandemic compared to the previous year.

**Methods:**

We retrospectively enrolled patients with soft tissue, bone sarcoma, and aggressive benign musculoskeletal diseases diagnosed during the pandemic (COVID group) or the year before (control group) at the IRCCS Regina Elena National Cancer Institute in Rome. Patients who had undergone a psychological assessment with the EORTC QLQ-C30 and the Distress Thermometer at diagnosis were included in the final analysis. We analyzed whether there is a difference in the various domains of quality of life between the two groups and whether there are changes over time in each group.

**Results:**

We enrolled 114 patients (72 control group; 42 COVID group), affected by soft tissue (64%), bone sarcoma (29%), and aggressive benign musculoskeletal diseases (7%). We did not observe significant differences in the health-related quality of life domains in the control and COVID groups, except for the financial domain (*p* = 0.039), with 9.7% vs. 23.8% of patients with a score > 0 in the control and COVID groups, respectively. We observed emotional distress at diagnosis in 48.6% of patients in the control group vs. 69.0% in the COVID group (*p =* 0.034). In the control group, we observed an improvement in physical function (*p =* 0.043) and in QoL (*p =* 0.022), while in the COVID group, we observed a deterioration in role function (*p =* 0.044) during follow-up. In the COVID group, 22.2% of patients were concerned about COVID-19, 61.1% by tumor, 91.1% stated that the pandemic worsened their subjective perception of cancer, and 19.4% perceived that their quality of care had worsened.

**Conclusion:**

We observed a higher level of distress among patients diagnosed during the pandemic compared to the year before, probably due to the increased concern for both infection and cancer, the worsened perception of health status, and the perception of a poorer quality of health care.

## 1. Introduction

At the end of 2019, a new Coronavirus, called Severe Acute Respiratory Syndrome Coronavirus 2 (Sars-CoV-2), emerged in China, leading to the development of a new disease, the COVID-19 (Zhu et al., 2020). In early 2020, the Sars-CoV-2 spread rapidly to the rest of the world leading to the declaration of a pandemic by the World Health Organization (WHO) on 11th March 2020 (WHO, 2020). To limit the contagion, many countries implemented a lockdown that limited interpersonal contacts and reduced work activities to only those that were strictly necessary. In this context, hospitals also underwent a reorganization of their activities concentrating their resources to cope with the ongoing health emergency (Onesti et al., 2020, 2021a).

With regard to cancer patients, literature data showed both a higher risk of contracting the infection and a higher risk of developing complications of the disease, especially for those with active disease and undergoing treatment (Liang et al., 2020; Yu et al., 2020). More than 30% of cancer patients during the first peak of the pandemic were more worried about COVID-19 than cancer and adopted all suggested preventive measures to avoid infection (Onesti et al., 2021b). Cancer patients were conscious to be at greater risk of complications and death, but disagreed with the possibility of either discontinuing or changing treatment protocols (Onesti et al., 2021b). A worse perception of general health status with emotional alterations mostly characterized by anxiety and loss of energy has been described by uro-oncologic patients after canceling surgery due to the COVID-19 outbreak (Greco et al., 2021). Another study conducted on 14 patients with various cancer types showed that anxiety, depressive symptoms, and moderate/high stress were experienced by cancer patients during the pandemic, mainly due to lifestyle changes and uncertainty over treatment schedules (Forner et al., 2021). Stress and adaptation disorders were observed about 40% more frequently among patients with a breast cancer diagnosis during the COVID-19 outbreak compared to patients with diagnoses performed during the years prior to the pandemic (Park et al., 2022). An increase in sleep disturbance and feelings of loneliness were observed during the pandemic, where the latter was associated with worsening depression, anxiety, and a higher level of stress (Bargon et al., 2021; Rentscher et al., 2021; Bethea et al., 2022). Beyond emotional distress, some studies have analyzed different items of health-related quality of life (HRQoL) patient reported outcomes, showing that functional aspects and symptoms were not significantly influenced by the pandemic (Koinig et al., 2021; Alexander et al., 2022).

Sarcomas are rare cancers accounting for less than 1% of malignancies, with an incidence of 4–5/100,000/year for soft tissue sarcomas (STS) and 0.8–0.9/100,000/year for bone sarcomas (BS) in Europe (Gatta et al., 2017; Gronchi et al., 2021; Strauss et al., 2021). They affect patients of all age groups, and often at a young age. They are very heterogeneous diseases that can originate at any site in the body, frequently leading to symptoms characterized by physical disabilities that impact the quality of life (QoL; Gronchi et al., 2021; Strauss et al., 2021). These aspects lead the patient toward both emotional distress and worsening of functional domains on QoL assessment scales. In addition, the rarity of the disease itself may induce greater insecurity in the patient. About half of the sarcoma patients showed a high level of psychological distress and a worse QoL especially in the elderly or in patients under active treatment (Horick et al., 2017; Bergerot et al., 2018). Distress and worsening of QoL appear early in sarcoma patients, with a nadir at the time of surgery, and persist for a long time, with recovery during follow-up (Tang et al., 2015; Maggi et al., 2019). In the context of the COVID-19 outbreak, a second stressful element was added. Data in literature showed that the COVID-19 pandemic had an impact on several aspects of functional activities in sarcoma patients, such as employment, finances, family and social life, emotional well-being, and feeling of loneliness (Younger et al., 2020). Patients that are receiving palliative treatment showed worse physical functioning, worse scores in HRQoL domains, and higher insomnia compared to sarcoma patients treated with curative intent during the pandemic (Younger et al., 2020). However, there is no data in the literature concerning either emotional distress or HRQoL in patients diagnosed with sarcoma during the pandemic vs. patients diagnosed before the pandemic. The impairment of quality of life and increased emotional distress in cancer patients, including sarcoma patients, is well known, but the impact that the pandemic had on this already stressful condition has not been adequately studied so far (Maggi et al., 2019, 2021).

In this report, we present data from a supplementary analysis of the SarCorD Study (*Sar*coma *Cor*onavirus diagnostic *D*elay), which is a retrospective analysis conducted at our Institute to assess diagnostic delay in patients with BS, STS, and aggressive benign musculoskeletal disease (ABMD; Onesti et al., 2022). The main findings were recently published showing a diagnostic delay due to the pandemic, which did not have an impact on start of treatment, survival, or stage at diagnosis. Moreover, we observed a reduction in the number of new admissions during the pandemic and a decrease in the number of new diagnoses, especially during the first trimester of the pandemic (Onesti et al., 2022). This additional analysis aims to assess whether there is a difference in HRQoL and in emotional distress in patients diagnosed during the pandemic compared to patients diagnosed within the previous 12 months. Moreover, we analyzed changes in QoL and distress over time in the two groups. When interpreting the results of this study, one must take into consideration that there were no specific restrictions on patients accessing our facility, but only for accompanying persons, as well as the use of personal protective equipment during the pandemic.

## 2. Materials and methods

We performed a single-center retrospective study including patients referred to the Multidisciplinary Sarcoma Outpatient Clinic of the IRCCS Regina Elena National Cancer Institute in Rome during the first 12 months of the COVID-19 pandemic and the 12 months prior. At our Institute, a European Reference Network on Rare Adult Cancers (EURACAN) centre with expertise in the domain of sarcomas and rare cancers, a prospective collection of all new cases of rare cancers, including sarcomas, has been carried out since January 2018. From this internal platform, the clinical cases object of this study were extracted (EURACAN, n.d.).

This study included patients who performed their first outpatient consultation at our Institute between 9th March 2019 and 8th March 2021 and with a histological diagnosis obtained/confirmed by the Pathological Anatomy Laboratory of our Institute regarding STS, BS, or ABMD (giant cell tumors of bone, aggressive fibromatosis, and pigmented villonodular tenosynovitis). We excluded patients having a malignancy different from those mentioned above or with a lack of clinical data. Of the 372 patients enrolled in the SarCorD study, we excluded patients who performed the first outpatient visit for reasons other than a new diagnosis and patients without data about HRQoL questionnaires from this secondary analysis.

Patients were classified into the control group or the COVID group according to the date of their first histological diagnosis performed between 9th March 2019 and 8th March 2020 or between 9th March 2020 (starting date of the National lockdown in Italy) and 8th March 2021, respectively.

The clinical data collected for the QoL analysis were: demographic data (age at diagnosis, sex, educational grade, professional occupation, marital status, childhood); histological diagnosis; extension of the disease; the type of first treatment; whether followed by psychological support; and results of HRQoL questionnaires.

HRQoL questionnaires were administered in person in the clinical practice setting during outpatient visits and retrospectively collected by a specialized psychologist with experience in cancer patient support. We collected data about two time points: the first is at first hospital admission for new diagnosis, and a second timepoint, that is (a) during the first access to the clinic during the pandemic (after 9th March 2020) for patients in the control group, or (b) at the end of the first oncological treatment for patients in the COVID group. The questionnaires administered were the European Organization for Research and Treatment of Cancer Quality of Life (EORTC QLQ-C30) and the Distress Thermometer (Distress Thermometer Tool Translations, n.d.; EORTC, n.d.). In particular, for the latter, the cut-off of 4 was considered to define patients as emotionally distressed (score > 4) or not emotionally distressed (score ≦ 4). In addition, at the time of the psychological assessment during the pandemic, from 9th March 2020 to 28th February 2022, during visits in the clinical practice setting patients were asked opinions concerning the delay in the various phases of diagnosis and treatment process, regarding COVID and cancer, personal perception of their illness, and of their quality of care as routine interview questions.

The study was conducted according to the Declaration of Helsinki and was approved by the Local Ethics Committee under the number 1676/22 and the acronym SarCorD Study. A signed informed consent was not required due to the retrospective nature of the study consistent with the current Institutional rules and National legislation.

### 2.1. Statistical analysis

We reported the categorical variables through absolute and relative frequencies, whereas the continuous variables through standard deviations (SD) or median values and interquartile range (IQR). Kolmogorov–Smirnov normality test was calculated for all the continuous variables. To explore the differences between continuous variables, we performed the Mann–Whitney or Student *T*-test, as appropriate. The relationships between categorical variables were analyzed using the Pearson’s Chi-square test. The temporal comparisons were analyzed by the Wilcoxon test. We used a univariate logistic regression model to identify variables that could have played a role in the risk of worse QoL. A value of *p* < 0.05 was considered statistically significant. Statistical analyses were carried out using SPSS version 21.0 (SPSS Inc., Chicago, Illinois, United States).

## 3. Results

### 3.1. Patients’ characteristics

Of the 372 patients enrolled in the SarCorD study, 56 were excluded because the histological diagnosis had been made before the set periods and 202 were excluded due to the absence of QoL evaluation questionnaires. Overall, 114 patients were eligible for the final QoL analysis. The patient characteristics included and excluded from the analysis are summarized in Table 1. Among the 114 patients included in the study, 72 belonged to the control group and 42 to the COVID group. The median age at diagnosis was 51.8 ± 16.6 years for the control group and 54.0 ± 14.2 years for the COVID group. Patients were male/female in 61.1%/38.9% and 57.1%/42.9% of the cases in the two groups, respectively. The disease was metastatic at diagnosis in 14.1% of the cases in the control group and in 12.2% in the COVID group. The most common diagnosis was STS in 61.1% and 69.0% of the cases in the two groups, followed by BS in 27.8% and 31.0% of the cases, and by ABMD in 11.1% and 0.0% of the cases, respectively. The first treatment received was a local treatment (surgery or radiotherapy) in most of the cases, accounting for 65.3% in the control group and 57.1% in the COVID group, followed by chemotherapy or chemoradiotherapy in 26.4% and 33.3% of the cases respectively, and by other treatments (electrochemotherapy or follow-up strategy) in 8.3 and 9.5% of the cases, respectively. A lower percentage of patients in the control group benefitted from psychological support compared to the COVID group (31.9% vs. 47.6%, respectively), although the difference was not statistically significant (*p* = 0.096). The two groups showed similar characteristics as reported in Table 2.

**Table 1:** Characteristics of patients enrolled in the SarCorD study and included/excluded from QoL analysis.

	Patients included in the analysis	Patients excluded from the analysis
	*N* = 114	*N* = 258
	*N* (%)	*N* (%)
Age (mean ± SD)	54.3 ± 16.57	58.4 ± 18.5
**Gender**		
M	66 (57.9)	162 (62.8)
F	48 (42.1)	96 (37.2)
**Educational level**		
Until first level of secondary school	34 (29.8)	32 (12.4)
Equal or upper than second level of secondary school	75 (65.8)	55 (21.3)
Unknown	5 (4.4)	171 (66.3)
**Occupational status**		
No	50 (43.9)	35 (13.6)
Yes	58 (50.9)	50 (19.4)
Unknown	6 (5.2)	173 (67.0)
**Civil status**		
Single/divorced/widowed	42 (36.8)	15 (5.8)
Married/cohabiting	62 (54.4)	58 (22.5)
Unknown	10 (8.8)	185 (71.7)
**Sons**		
No	23 (20.2)	34 (13.2)
Yes	62 (54.4)	77 (29.8)
Unknown	29 (25.4)	147 (57.0)
**Extension of the disease**		
Localized	97 (85.1)	198 (76.7)
Metastatic	15 (13.2)	32 (12.4)
Unknown	2 (1.7)	28 (10.9)
**Hystotype**		
Soft tissue sarcoma/Dermatofibrosarcoma protuberans	72 (63.2)	164 (63.6)
Bone sarcoma/Ewing Sarcoma	33 (28.9)	50 (19.4)
Giant cell tumor of bone/Benign muscoloscheletal aggressive disease	8 (7.0)	21 (8.1)
Gastrointestinal stromal tumor/Kaposi sarcoma	1 (0.9)	23 (8.9)

**Table 2:** Patients’ characteristics in control and COVID group.

*N* = 114	Control group	COVID group	Chi-Square test
	*N* = 72	*N* = 42	*p-*value
	*N (%)*	*N (%)*	
Age (mean ± SD)	51.8 ± 16.6	54.0 ± 14.2	0.473^*^
**Gender**			
M	44 (61.1)	24 (57.1)	0.677
F	28 (38.9)	18 (42.9)	
**Educational level**			
Until first level of secondary school	22 (30.6)	12 (28.6)	0.64
Equal or upper than second level of secondary school	45 (62.5)	30 (71.4)	
Unknown	5 (6.9)	0 (0.0)	
**Occupational status**			
No	32 (44.4)	18 (42.9)	0.567
Yes	34 (47.2)	24 (57.1)	
Unknown	6 (8.3)	0 (0.0)	
**Civil status**			
Single/divorced/widowed	27 (37.5)	15 (35.7)	0.636
Married/cohabiting	37 (51.4)	25 (59.5)	
Unknown	8 (11.1)	2 (4.8)	
**Sons**			
No	13 (18.1)	10 (23.8)	0.995
Yes	35 (48.6)	27 (64.3)	
Unknown	24 (33.3)	5 (11.9)	
**Type of first treatment**			
Chemotherapy/chemoradiotherapy	19 (26.4)	14 (33.3)	0.682
Local treatment	47 (65.3)	24 (57.1)	
Other	6 (8.3)	4 (9.5)	
**Extension of the disease**			
Localized	61 (84.7)	36 (85.7)	0.777
Metastatic	10 (13.9)	5 (11.9)	
Unknown	1 (1.4)	1 (2.4)	
**Hystotype**			
Soft tissue sarcoma/Dermatofibrosarcoma protuberans	43 (59.7)	29 (69.0)	0.127
Bone sarcoma/Ewing Sarcoma	20 (27.8)	13 (31.0)	
Giant cell tumor of bone/Benign muscoloskeletal aggressive disease	8 (11.1)	0 (0.0)	
Gastrointestinal stromal tumor/Kaposi sarcoma	1 (1.4)	0 (0.0)	

* Student’ *T*-test.

### 3.2. Quality of life and distress in patients diagnosed during the pandemic vs. control group

We did not observe significant differences between the control group and the COVID group, for both functional and symptomatic domains from the EORTC QLQ-C30 instrument at diagnosis, except for the financial domain (Table 3). We observed a different distribution for financial difficulties scores (*p* = 0.039), with a median score of 0.00 (IQR 0.00–0.00) for the control group vs. 0.00 (IQR 0.00–8.25) for the COVID group and a mean score of 4.15 (SD 13.63) vs. 11.10 (SD 21.71) in the two groups, respectively. Overall, most patients have a score of 0 in both groups, while only 9.7% of patients (7 out of 72) in the control group and 23.8% (10 out of 42) in the COVID group had a score > 0 (*p* = 0.057). No demographic factors (age, sex, marital status, occupational status, educational level, childhood) or factors related to the disease (histology, extension of the disease, type of treatment) influenced the financial distress domain.

**Table 3:** Mean score (±SD) and median scores (IQR) in the EORTC QLQ-C30 domain.

EORTC QLQ-C30 domain		Control group		COVID group		*P*-value (Mann Whitney test)
		Mean (SD)	Median (IQR)	Mean (SD)	Median (IQR)	
Functional scales	Physical function	77.03 (23.89)	83.00 (67.00–93.00)	69.33 (28.92)	80.00 (52.25–100.00)	0.234
	Role function	71.92 (30.22)	83.00 (67.00–100.00)	71.41 (33.59)	83.00 (50.00–100.00)	0.693
	Emotional function	77.46 (18.23)	83.00 (67.00–92.00)	71.83 (23.31)	83.00 (58.00–92.00)	0.332
	Cognitive function	93.01 (13.36)	100.00 (83.00–100.00)	93.62 (12.18)	100.00 (83.00–100.00)	0.860
	Social function	90.90 (16.56)	100.00 (83.00–100.00)	82.95 (29.79)	100.00 (67.00–100.00)	0.611
	Quality of Life	68.88 (19.37)	75.00 (50.00–83.00)	68.52 (25.21)	83.00 (50.00–83.00)	0.644
Symptomatic scales	Dyspnea	1.37 (6.64)	0.00 (0.00–0.00)	5.28 (15.98)	0.00 (0.00–0.00)	0.116
	Pain	18.01 (20.48)	17.00 (0.00–33.00)	20.64 (24.08)	17.00 (0.00–33.00)	0.688
	Fatigue	15.93 (17.71)	11.00 (0.00–33.00)	21.10 (26.88)	5.50 (0.00–33.00)	0.654
	Insomnia	17.93 (20.06)	0.00 (0.00–33.00)	21.36 (26.38)	0.00 (0.00–33.00)	0.733
	Loss of appetite	2.31 (10.19)	0.00 (0.00–0.00)	4.73 (13.88)	0.00 (0.00–0.00)	0.230
	Nausea	1.18 (4.35)	0.00 (0.00–0.00)	4.76 (12.88)	0.00 (0.00–0.00)	0.165
	Constipation	4.13 (10.99)	0.00 (0.00–0.00)	7.91 (20.55)	0.00 (0.00–0.00)	0.483
	Diarrhea	0.00 (0.00)	0.00 (0.00–0.00)	0.00 (0.00)	0.00 (0.00–0.00)	1.000
	Financial difficulties	4.15 (13.63)	0.00 (0.00–0.00)	11.10 (21.71)	0.00 (0.00–8.25)	**0.039**

SD, Standard Deviation; IQR, interquartile range. Mean and median scores of the EORTC QLQ-C30 domains. The domains are divided into functional scales, for which a higher score corresponds to a better function, and symptomatic scales, for which the lower score corresponds to the absence of symptoms. Bold values indicated statistically significant results.

We observed a significantly lower rate of patients with high emotional distress at diagnosis in the control group (48.6%) vs. the COVID group (69.0%; *p = 0.034*). In this case, too, we did not observe any influence by demographic or cancer-related factors.

### 3.3. Changes in quality of life and distress during the pandemic

There were 36 evaluable patients for the control group and 42 for the COVID group at a second time point during the pandemic. The proportion of patients that benefited from psychological support is 36.1% and 47.6% in the control and COVID groups, respectively (*p* = 0.305). Some patients had not completed the questionnaires at the second time point, due to discontinuing psychological counseling, refusal by the patient, loss at follow-up, or death. The median time intervals between the two psychological evaluations were of 15 months (range 4–29) for the control group and of 6 months (range 1–19) for the COVID group.

For patients diagnosed before the pandemic (control group), we observed an improvement in the physical function and QoL domains during the pandemic compared to the diagnosis time point, with a value of *p* of 0.043 and 0.022, respectively (Table 4). In this group of patients, we observed a non-significant (*p* = 0.248) impairment of emotional distress from 40% of patients showing a high level of distress at diagnosis vs. 51.4% at follow-up during the pandemic.

**Table 4:** Changes in EORTC QLQ-C30 domains from diagnosis to the pandemic time point for the control group.

EORTC QLQ-C30 Domain		Diagnosis timepoint		Pandemic timepoint		*P*-value (Mann Whitney test)
		Mean (SD)	Median (IQR)	Mean (SD)	Median (IQR)	
Functional scales	Physical function	73.14 (28.47)	87.00 (61.75–93.00)	82.22 (21.29)	87.00 (80.00–100.00)	**0.043**
	Role function	63.83 (33.98)	87.00 (41.50–100.00)	76.89 (30.84)	83.00 (67.00–100.00)	0.139
	Emotional function	78.03 (19.73)	100.00 (83.00–100.00)	80.22 (17.94)	83.00 (75.00–92.00)	0.726
	Cognitive function	92.53 (12.27)	100.00 (83.00–100.00)	90.28 (18.35)	100.00 (83.00–100.00)	0.885
	Social function	92.06 (18.06)	100.00 (83.00–100.00)	93.86 (12.15)	100.00 (87.25–100.00)	0.850
	Quality of Life	67.92 (22.26)	83.00 (50.00–83.00)	76.78 (18.76)	83.00 (67.00–83.00)	**0.022**
Symptomatic scales	Dyspnea	0.92 (5.50)	0.00 (0.00–0.00)	2.75 (9.25)	0.00 (0.00–0.00)	0.317
	Pain	18.03 (21.59)	8.50 (0.00–33.00)	17.58 (20.27)	17.00 (0.00–33.00)	0.952
	Fatigue	15.81 (19.08)	11.00 (0.00–33.00)	16.75 (20.39)	11.00 (0.00–33.00)	0.842
	Insomnia	0.00 (21.78)	0.00 (0.00–33.00)	12.86 (18.21)	0.00 (0.00–33.00)	0.106
	Loss of appetite	2.78 (12.31)	0.00 (0.00–0.00)	4.61 (14.13)	0.00 (0.00–0.00)	0.577
	Nausea	0.47 (2.83)	0.00 (0.00–0.00)	6.03 (19.17)	0.00 (0.00–0.00)	0.109
	Constipation	6.42 (13.25)	0.00 (0.00–0.00)	8.33 (20.17)	0.00 (0.00–0.00)	0.339
	Diarrhea	0.00 (0.00)	0.00 (0.00–0.00)	1.83 (7.67)	0.00 (0.00–0.00)	0.157
	Financial difficulties	3.69 (13.28)	0.00 (0.00–0.00)	4.61 (14.13)	0.00 (0.00–0.00)	0.783

SD, Standard Deviation; IQR, interquartile range. Mean and median scores of the EORTC QLQ-C30 domains at diagnosis and during the pandemic for patients in the Control group. The domains are divided into functional scales, for which a higher score corresponds to a better function, and symptomatic scales, for which the lower score corresponds to the absence of symptoms. Bold values indicated statistically significant results.

For patients diagnosed during the pandemic (COVID group), we observed a deterioration of the role domain during follow-up (*p* = 0.044; Table 5). In this group of patients, we observed a non-significant improvement of emotional distress from diagnosis to follow-up time point, with 69% of patients showing high distress at diagnosis during the pandemic vs. 56.7% of patients with high distress at follow-up visits still performed during the pandemic (*p* = 0.109).

**Table 5:** Changes in EORTC QLQ-C30 domains from diagnosis to the pandemic timepoint for the COVID group.

EORTC QLQ-C30 Domain		Diagnosis timepoint		Pandemic timepoint		*P*-value (Mann Whitney test)
		Mean (SD)	Median (IQR)	Mean (SD)	Median (IQR)	
Functional scales	Physical function	69.33 (28.92)	80.00 (52.25–93.00)	66.55 (25.40)	70.00 (45.25–88.50)	0.543
	Role function	71.41 (33.59)	83.00 (50.00–100.00)	67.00 (39.13)	67.00 (17.00–100.00)	**0.044**
	Emotional function	71.83 (23.31)	83.00 (58.00–92.00)	71.95 (22.27)	75.00 (58.00–92.00)	0.900
	Cognitive function	93.62 (12.18)	100.00 (100.00–100.00)	93.19 (13.36)	100.00 (100.00–100.00)	0.740
	Social function	82.95 (29.79)	100.00 (100.00–100.00)	86.91 (23.97)	100.00 (100.00–100.00)	0.628
	Quality of Life	68.52 (25.21)	83.00 (50.00–83.00)	69.91 (17.73)	67.00 (67.00–83.00)	0.263
Symptomatic scales	Dyspnea	5.29 (15.98)	0.00 (0.00–0.00)	4.71 (11.69)	0.00 (0.00–0.00)	1.000
	Pain	20.64 (24.08)	17.00 (0.00–33.00)	22.62 (24.08)	17.00 (0.00–33.00)	0.361
	Fatigue	21.10 (26.88)	5.50 (0.00–33.00)	27.95 (23.52)	22.00 (11.00–44.00)	0.060
	Insomnia	21.36 (26.38)	0.00 (0.00–0.00)	26.93 (27.85)	33.00 (0.00–33.00)	0.093
	Loss of appetite	4.74 (13.88)	0.00 (0.00–0.00)	3.95 (13.16)	0.00 (0.00–0.00)	0.783
	Nausea	4.76 (12.88)	0.00 (0.00–0.00)	3.57 (11.94)	0.00 (0.00–0.00)	0.438
	Constipation	7.90 (20.55)	0.00 (0.00–0.00)	7.14 (21.53)	0.00 (0.00–0.00)	1.000
	Diarrhea	0.00 (0.00)	0.00 (0.00–0.00)	1.60 (10.34)	0.00 (0.00–0.00)	0.317
	Financial difficulties	11.10 (21.71)	0.00 (0.00–8.25)	15.83 (29.66)	0.00 (0.00–33.00)	0.223

SD, Standard Deviation; IQR, interquartile range. Mean and median scores of the EORTC QLQ-C30 domains at diagnosis and during the pandemic for patients in the COVID group. The domains are divided into functional scales, for which a higher score corresponds to a better function, and symptomatic scales, for which the lower score corresponds to the absence of symptoms. Bold values indicated statistically significant results.

### 3.4. Subjective perception of disease and quality of care during the pandemic

Among the patients interviewed during the pandemic, none in the control group vs. 12.5% in the COVID group stated that they had delayed access to their first consultation. Access to radiological investigations was delayed by 9.7% and 5.1% of patients in the two groups, respectively. Biopsy was delayed by none of the patients in the control group and 6.1% of patients in the COVID group, while surgery was delayed by 3.2% and 2.9% of patients in the two groups, respectively. No patient reported having delayed systemic medical treatment. Follow-up visits were not performed regularly by 3.2% and 2.6% of patients in the control and COVID groups, respectively.

During the pandemic, 16.7% of patients in the control group stated that they were concerned about COVID-19 vs. 22.2% of patients in the COVID group. In addition, among patients in the control group, concern about cancer was lower than in the COVID group, with 40% and 61.1% of patients saying that they were worried about cancer in each group, respectively. Most patients, 100% in the control group and 91.9% in the COVID group stated that the pandemic worsened their subjective perception of cancer.

The quality of healthcare during the pandemic was perceived as impaired by 10% of the patients in the control group and 19.4% in the COVID group.

## 4. Discussion

In this study, we investigated the various domains of QoL using the EORTC QLQ-C30 questionnaire and the psycho-emotional aspects through adopting the Distress Thermometer in patients diagnosed during the first year of the pandemic and the year prior. Data in literature showed a worse perception of conditions in general, anxiety, depressive symptoms, sleep disturbance, feelings of loneliness, and loss of energy in cancer patients during the pandemic (Bargon et al., 2021; Forner et al., 2021; Greco et al., 2021; Rentscher et al., 2021; Bethea et al., 2022). To our knowledge not much data are available regarding distress and QoL in patients diagnosed during the pandemic. Park et al. observed a higher rate of stress and adaptation disorders among patients with a breast cancer diagnosed during the COVID-19 outbreak compared to patients with diagnoses performed before the pandemic (Park et al., 2022). So far, no similar data are available in the literature in sarcoma patients and our study is the first report on this group of patients. We observed no significant differences in the functional and symptomatic scales of the questionnaire, except for a different distribution of scores regarding financial difficulties, with more patients stating to have financial issues when diagnosed during the pandemic. This finding is clearly understandable considering that the various items on the EORTC QLQ-C30 deal with aspects such as symptoms or physical function, cognitive function, and so on, which are directly influenced by cancer. It is different for the domain of financial distress, which can be modified due to the enforced work interruption under lockdown, observed especially in the first phase of the pandemic. However, the observed difference is minimal, probably due to the small sample size of our cohort. Further studies on larger sample sizes are needed to confirm this result. On the other hand, we observed an increase in emotional distress in patients diagnosed during the first year of the pandemic, with patients having a high level of emotional distress about 20% higher than patients diagnosed before the COVID-19 outbreak. Neither demographic nor tumor-related factors played a role in the increase in distress. Interestingly, a higher rate of patients diagnosed during the pandemic benefited from psychological support compared to patients diagnosed before the pandemic. The above-mentioned findings may be driven by the fact that these patients were concerned not only about the tumor but also about COVID-19, with approximately 20% of them concerned about the infection, about 20% higher rate of patients worried about cancer compared to patients diagnosed before the pandemic, and almost all patients who stated that the pandemic worsened their subjective perception of cancer. In addition, 10% of patients in the control group and about 20% in the COVID group said they perceived that the quality of care had reduced because of the pandemic. Another factor that may have had a negative effect on psycho-emotional distress is the delay in the various stages of patient care. In fact, data from the primary objective analysis of the SarCorD study, of which this sub-analysis is part of, showed a diagnostic delay in sarcoma patients during the pandemic, although this delay did not have an impact on the onset of treatment (Onesti et al., 2022).

This study also analyzed the change in QoL and emotional distress over time. This analysis has been done in each group separately and the results are not comparable. In the control group, we observed improved scores for physical function and QoL during follow-up, even though the differences are very small. This finding is consistent with data in literature showing recovery time after treatment of a localized sarcoma (Maggi et al., 2019). This difference could also be attributed to the fact that people in poor clinical conditions did not participate in the second survey as well as to the fact that part of the patients received psychological support. In the COVID group, we observed worsening scores in the role domain, which assesses the patient’s ability to perform his/her work, housework, and hobbies. This is probably related to the care pathway accomplished entirely during the pandemic, which therefore had a negative impact on patients’ activity, due to the wide introduction of working from home, especially for frail individuals, as well as to a reduction in social activities.

To our knowledge, this is the first study that analyzed QoL and psycho-emotional distress in patients who were diagnosed with STS, BS, or ABMD during the pandemic vs. patients who received the same diagnosis prior the pandemic. This study has some limitations, such as its retrospective nature, the non-standardized timing of the follow-up questionnaires, and the small sample size. Furthermore, the results presented in this article are based on a secondary analysis of a study not designed with the objective of assessing QoL. Therefore, the results obtained need to be confirmed with larger studies designed with the aim of assessing QoL in sarcoma patients diagnosed during the COVID-19 period or prior. However, the results obtained provide the basis for further studies of this type, both multicenter retrospective and prospective, to assess the impact of the pandemic in the long term. Moreover, from a clinical perspective, these results highlighted emotional distress, which requires more attention in the comprehensive care of the patient, also from a psychological point of view, especially during such a particular period like the pandemic.

In conclusion, we observed a higher level of emotional distress among patients who were diagnosed with STS, BS, or ABMD during the first year of the pandemic than among those who were diagnosed in the previous year, whereas there was no impact on either functional QoL scales or symptoms, except for financial difficulties. These results may be explained by several factors, such as the increased concern during the pandemic for both infection and cancer, worsened perception of their health status due to the pandemic, perception of a poorer quality of healthcare, and delay in the various stages of the diagnostic-therapeutic pathway. Greater attention toward the psychological aspects of cancer patients is important in the presence of other stressful conditions, such as the COVID-19 pandemic. Close psychological support must be considered in the presence of possible similar scenarios as well as in the presence of several concomitant stressors.

## Data availability statement

The original contributions presented in the study are included in the article/supplementary material, further inquiries can be directed to the corresponding author.

## Ethics statement

The studies involving human participants were reviewed and approved by Regina Elena Ethics Committee. Written informed consent for participation was not required for this study in accordance with the national legislation and the institutional requirements.

## Author contributions

CO, SV, BR, MC, VF, and GM conceived the study. CO, BR, FSp, MC, AC, and VF wrote the project. CO, FN, GM, DM, and EC were involved in data collection. FSp performed statistical analysis. CO, SV, BR, RB, GC, VF, and GM interpreted the results. CO wrote the article. All authors contributed to the article and approved the submitted version.

## Funding

This work was financially supported through funding from the institutional “Ricerca Corrente 2023” granted by the Italian Ministry of Health.

## Conflict of interest

The authors declare that the research was conducted in the absence of any commercial or financial relationships that could be construed as a potential conflict of interest.

## Publisher’s note

All claims expressed in this article are solely those of the authors and do not necessarily represent those of their affiliated organizations, or those of the publisher, the editors and the reviewers. Any product that may be evaluated in this article, or claim that may be made by its manufacturer, is not guaranteed or endorsed by the publisher.

## Abbreviations

WHO: World Health Organization
HRQoL: Health-related quality of life
QoL: Quality of life
STS: Soft tissue sarcoma
BS: Bone sarcoma
ABMD: Aggressive Benign Muscoloskelatal Disease
EURACAN: European Reference Network on Rare Adult Cancers
EORTC QLQ-C30: European Organization for Research and Treatment of Cancer Quality of Life
SD: Standard deviation
IQR: Interquartile range
